# Utilizing social foraging as a framework to study decisions in groups

**DOI:** 10.1016/j.isci.2026.114940

**Published:** 2026-02-12

**Authors:** Ketika Garg, Wenning Deng, Dean Mobbs

**Affiliations:** 1 Department of Humanities and Social Sciences and Computation, California Institute of Technology, 1200 E California Blvd, HSS 228-77, Pasadena, CA 91125, USA; 2 Neural Systems Program at the California Institute of Technology, 1200 E California Blvd, HSS 228-77, Pasadena, CA 91125, USA

**Keywords:** Neuroscience, Cognitive neuroscience, Social sciences

## Abstract

A central goal of the behavioral sciences is to understand how individuals decide between rewarding and conflicting options. Foraging theory, which is rooted in ecology and evolutionary theory, has helped advance this pursuit but has largely focused on individual decision-making processes. In this article, we extend beyond individual agents and propose social foraging as a promising avenue to study social decisions. We synthesize key socio-cognitive elements of social interactions that are particularly amenable to study through foraging paradigms, such as social inference, coordination, and collective behavior, especially in humans. We then propose a social foraging framework that distinguishes between the asocial and social components involved in the decision-making process and describes how the integration of these components drives decisions in social foraging. Our framework bridges research across disciplines to provide a promising new avenue for the study of social behavior by linking decisions across different scales, from individuals to collectives.

## Graphical abstract

**Figure:**
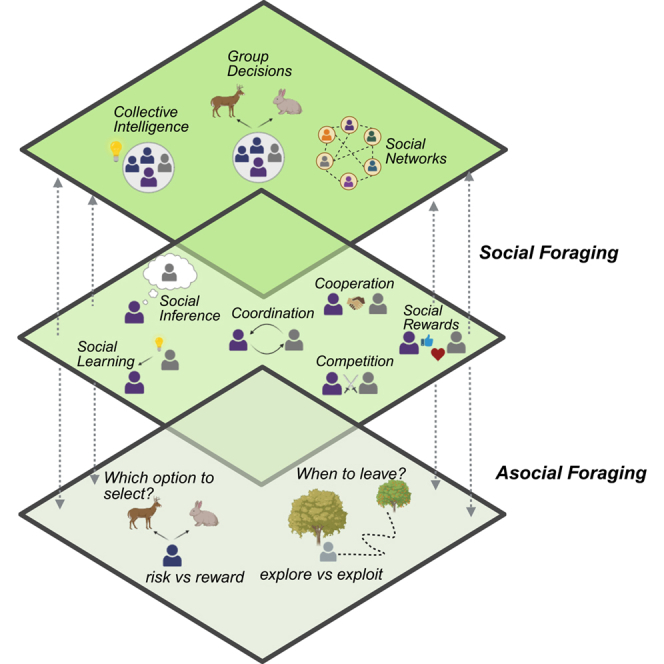

## Introduction

Sociality has been a defining feature in our species’ history and success. From the beginning of our time as hunter-gatherers to the present day, we rarely make our decisions in isolation. Instead, we make decisions as part of a group and even collectively as a group. Foraging theory has provided a framework for studying and situating human decision-making within ecological and evolutionary contexts.^1^ However, its applications have been largely limited to individuals and have overlooked the social aspects of decisions, such as social inference, social learning, coordination, and collective behavior. In this article, we introduce a social foraging framework that can help extend decision-making beyond the individual and toward a richer understanding of behavior and decision-making in groups, especially in naturalistic settings.

Since the early theories of optimal foraging,^2^^,^^3^^,^^4^ foraging paradigms have been a staple across the behavioral sciences. Optimal foraging models provide a naturalistic and ecologically valid context to examine decision-making under conditions that test an individual’s ability to balance costs and benefits to maximize their energy intake.^1^ They also provide tractable ways to simulate different conditions like uncertainty, threat, and risk, which induce fundamental trade-offs that underlie many decisions. For example, many foraging studies have investigated how individuals balance trade-offs between exploration and exploitation,^5^^,^^6^^,^^7^ speed and accuracy,^8^^,^^9^ and risk and safety^10^ to maximize their foraging success. Such trade-offs are often shaped by environmental conditions, state-dependent factors, and individual traits. Many perspectives on human and non-human foraging have drawn upon these core principles from behavioral ecology to characterize how individuals balance trade-offs in naturalistic decision-making.^1^^,^^11^^,^^12^^,^^13^^,^^14^^,^^15^

Humans and many other species forage in groups and engage in competition, collaboration, or a combination thereof. Compared with solitary foraging, foraging in groups poses new challenges such as coordination and competition, while giving rise to opportunities such as risk dilution and resource sharing. These phenomena and social dilemmas intertwine with existing foraging trade-offs and guide an individual forager’s decisions. Decision-making in a social context may also involve inferring others’ beliefs or intentions, integrating those with one’s preferences,^16^^,^^17^ guiding others’ decisions,^18^ and learning from others, often in a flexible and dynamic way.^16^^,^^19^ The type of social interactions, e.g., competitive or cooperative, can further modify these social decisions and the underlying computations.

In the broader context of evolution, social foraging has arguably been a key driver of social intelligence and general cognition^20^ and a primary contributor to the evolution of sociality. Social foraging may have even acted as a selective pressure on several cognitive processes like joint attention, social learning, and planning, all of which are crucial for navigating complex social environments.^21^ Social foraging occupies a special place in the context of human evolution.^22^^,^^23^^,^^24^ Successful collaboration and the ability to achieve goals by coordinating with others are considered as significant steps in human evolution. Social foraging also likely facilitated human socio-cognitive “niche,” i.e., the social and cognitive features or adaptations to suit social foraging needs, such as cooperation, coordination, theory of mind, and communication.^25^ For example, ethnological records from hunter-gatherer communities suggest widespread cooperation and division of labor between group members, where foraging parties take different routes in their daily foraging and bring back the collected food to a central camp to be shared by everyone. However, frequent coordination and cooperation hinge on cognitive adeptness in inferring others’ intentions and willingness to cooperate, as well as on social mechanisms, such as norms, that regulate cooperation and prosocial tendencies.^23^

Social decisions have been studied through diverse methodological approaches across disciplines. What these approaches lack is a framework that integrates multiple dimensions of social behavior in ecologically valid settings. In the context of psychology and cognitive neuroscience, we propose that social foraging paradigms can provide such a framework by incorporating naturalistic environments,^26^ where various trade-offs must be balanced simultaneously, and by integrating social and non-social components of decisions that are often studied separately. Social foraging builds upon general decision-making, where a sequence of decisions guides the forager toward a certain goal, while adding a layer of social processes. It also naturally extends to group-level processes, because foragers can act collectively, enabling the study of decision-making across multiple scales, from individuals to groups. We begin by reviewing different conceptual models and types of social interactions that have been studied within social foraging, drawing from established theories in behavioral ecology. We then outline the key socio-cognitive components of social interactions that underlie or manifest in social foraging, with a focus on human social foraging. We conclude by putting forward an integrative framework that contextualizes these components into a decision-making framework, while distinguishing between the asocial and social components of social decisions in foraging and illustrating how they can interact and inform each other. Our framework can open new avenues for the study of social and collective decisions, help compare them with individual decisions, and discover how they integrate with one another.

## Two key contexts of social foraging

**Figure 1:**
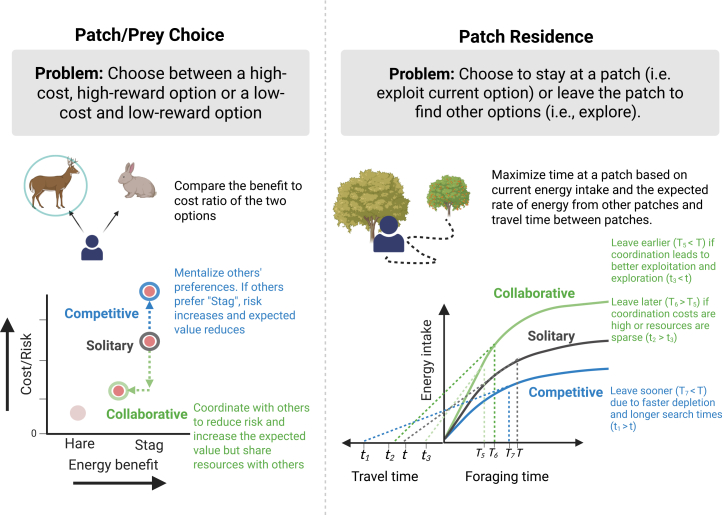
Social modifications to classical solitary foraging paradigms In solitary foraging, prey-choice and patch-residence decisions are driven by cost-benefit trade-offs and diminishing returns. In social foraging, these are shaped by others’ preferences, and the social context. In the prey-choice model, social context alters risk: under competition, risk increases; under collaboration, coordination can reduce risk and boost the expected value, but the energy benefit must be shared among group members. In the patch-residence model, social foraging can enhance exploitation and exploration via the sharing of information about the environment but may also introduce coordination costs or competition, leading to shorter or longer patch times depending on the social and physical conditions.

To survive in the natural world, an individual forager needs to decide which resources to consume and how to optimally search for resources, while avoiding dangers (e.g., predation or starvation risk). Patch-choice^27^ and patch-residence models^2^ are two canonical models in O*ptimal Foraging Theory* for examining individual foraging decisions^28^ (Figure 1). Patch (or prey) choice models evaluate costs (e.g., risk or effort) and benefits (e.g., energy or reward) associated with each option available. Patch-residence models add a temporal dimension to the decisions and predict when to leave a patch based on the current patch quality, expected future rewards, and the costs of harvesting or finding them (formalized by the marginal value theorem).

The natural world is riddled with different forms of social foraging, from quorum-sensing bacteria^29^ and eusocial insects^30^^,^^31^ to hunter-gatherers.^32^ We conceptualize social foraging as a phenomenon where individuals forage in each other’s presence and are influenced by other individuals. The presence of other foragers can create new phenomena such as competition, collaboration, and cooperation that are uniquely social. This social layer introduces new trade-offs and modifies what counts as an optimal decision.^33^ On the one hand, if competing for limited resources, social foraging can be detrimental to an individual’s survival. On the other hand, foraging with others can unlock a suite of benefits and possibilities unattainable by a single forager, such as safety against predators (e.g., risk dilution and protection), collective pooling of information, distributed energy expenditure, and division of tasks.^34^^,^^35^ Because each individual’s success depends on others’ actions, social foraging continually reshapes the underlying payoff landscape, making others’ actions central to determining what is considered “optimal” (see Box 1 for more details).

Box 1 **Game theory, social decisions, and social foraging**

Social foraging and social decision-making literature have shared roots in game theory.^36^ Traditionally, it has been useful to invoke game theory when individual fitness (or performance) is dependent on the actions of other individuals in a group.^37^ When viewed as a “game,” different strategies or actions can lead to different payoffs, and simulating games can help explore the interdependence of strategies. In behavioral economics and psychology, economic games have been widely used to study coordination, cooperation, selfish behavior, and social preferences, leading to the development of many standardized two-player games like Trust Game, Hawk-Dove Game, Prisoner’s Dilemma, and Stag Hunt. Other games, like the public goods game, extend beyond 2-person interactions and study how a person strategically interacts within a group.

Similar dynamics occur in social foraging, too, because of an inherent interdependence between an individual and others’ decisions.^38^ Many economic games are indeed an abstraction of foraging economics. For example, the stag-hunt game is a simplified hunting dilemma: two people need to coordinate to hunt for a bigger reward and miscoordination leads to a loss.^39^ Game-theoretic models offer equilibrium predictions to both collaborative and competitive contexts. For example, in the producer-scrounger game, optimal behavior is frequency dependent, i.e., it is better to choose the action not selected by others, which can lead to a mixed equilibrium in the group (co-existence of both strategies). In the stag-hunt game, both players hunting for stags or both hunting for hares are two pure strategy equilibria, where the optimal action is to match partners. Social foraging paradigms can build upon game-theoretic models by situating economic games in naturalistic settings to gain data beyond what two-choice paradigms can allow (e.g., movement data, synchronization, multi-agent coordination, threat). They can help create dynamic, continuous tasks that probe state-dependent actions^40^^,^^41^^,^^42^^,^^43^, and deviations from expected or “rational” behavior.

In this section, we outline two key contexts of social interactions particularly relevant in foraging: competitive and collaborative. Each of these contexts introduces trade-offs that do not exist in individual foraging. Yet, each context builds upon individual foraging decisions like finding food, selecting between patches, optimizing the time spent on a patch, and avoiding predators. We discuss a few models and approaches that have been central to understanding social foraging interactions. Given the complexity of social foraging, many of these models are simplifications of real-world behavior. In Figure 1, we provide an illustrative example of how predictions from classical foraging models in optimal foraging theory can be modified given these two contexts.

### Competitive social interactions

Competition shapes decisions in many social settings. In the face of limited resources, individuals often compete for them, and an individual’s success suffers in the presence of others. The level of competition between individuals can be affected by the foraging environment, such as resource types, availability, and distribution. To be successful under competition, individual foragers may need to predict others’ intentions and actions and adjust trade-offs to account for the presence and behavior of others. For instance, given the option to choose between two patches, an optimal solitary forager will prefer the one with more resources. However, in the presence of others, the level of competition at the two patches would influence the optimal decision (Figure 1).

Models like ideal free distribution predict the optimal distribution of foragers among resource patches to minimize competition while maximizing foraging efficiency.^44^ Studies have shown that when deciding between two patches or rewards, humans tend to choose options that minimize competition and maximize rewards.^45^^,^^46^^,^^47^^,^^48^^,^^49^ However, other constraints like the availability of information about resources can affect the level of attraction or repulsion between foragers^50^ and drive deviations from the ideal-free distribution model.^51^

Competitors’ actions dynamically reshape other individuals’ payoffs. *Game-theoretic models* have helped in predicting the optimality of an option depending on what actions or roles others are undertaking (Box 1). A canonical example is the *producer-scrounger* game.^36^^,^^52^ Producers discover food sites, implicitly broadcasting their location, whereas scroungers exploit these discoveries without paying the search cost. But if everyone chooses to be a scrounger, then the benefit of scrounging diminishes due to a lack of discoveries. The optimal action in this game is thus frequency dependent (i.e., based on how many others are choosing the same option): if there are many producers in the group, it is better to scrounge, but if there are too many scroungers, then it is better to be a producer. An optimal strategy (or the *evolutionarily stable strategy*) can emerge that maintains enough producers to balance the costs of searching against the benefits of scrounging. Other environmental features, like resource abundance, group size, and search strategies, can shift the optimal frequency of a strategy.^53^^,^^54^^,^^55^^,^^56^

### Collaborative social interactions

We define collaborative interactions as an umbrella term where two or more individuals can benefit from each other’s success. They may share some or all goals or coordinate their actions to produce mutually beneficial outcomes.^57^ When individuals forage in groups, advantages can arise as a by-product of each person maximizing their own energy returns, even in the absence of active collaboration.^58^ At a minimal level, group presence confers the benefit of *risk dilution*—the reduced probability of being targeted by a predator simply by virtue of being among others. This perceived reduction in personal risk can downregulate fear,^59^ reduce threat monitoring,^60^ and encourage decisions to join others despite competition.^10^

Beyond risk dilution, foraging with others can yield by-product gains via *information sharing*, more thorough exploitation of current patches, and more effective exploration of alternatives.^61^^,^^62^^,^^63^ In solitary foraging, foragers optimize search time, resources, and patch-residence times by trading off effort, time, uncertainty, and predation risk.^2^^,^^6^^,^^64^^,^^65^ In collaborative social foraging, decisions regarding patch residence or patch selection are influenced by the additional benefits of collective exploration/exploitation and by the costs of coordination, conditioned on others’ actions and the expected gains from a patch or prey upon sharing.

Decisions to be actively prosocial or to actively cooperate with others can lead to greater benefits, especially when individuals pool their efforts, energy, and resources under reciprocal interactions. While risk dilution is a by-product advantage of foraging with others, individuals can work together to collectively defend themselves against other groups or predators or coordinate to be jointly vigilant. Experiments on foraging under threat in humans have shown that individuals attend less to threats in a group and become collectively vigilant.^66^ Similar to the producer-scrounger dynamic, the optimal actions in a collaborative context are likewise frequency-dependent, with the optimal action being based on how many others have adopted the same strategy.^66^ Individual decisions between foraging and staying vigilant (based on the current energetic needs and others’ vigilance) can lead to the emergence of collective vigilance without explicit coordinating.^67^ Moreover, many collaborative dilemmas often map onto coordination games where performing an action is profitable only if others choose to do so (in a *stag-hunt analog*, a high-value prey is profitable only if partners commit simultaneously) (Box 1).

While some instances of coordination may emerge through simpler cognitive processes and heuristics,^68^^,^^69^ active and effective coordination can require predicting others’ behavior.^70^ It often depends on complex processes such as predicting others’ actions, social learning, joint intentionality, division of tasks, and social incentives. Compared to searching for multiple, static rewards, hunting for a single, mobile resource requires more complex inferences, coordination, and role differentiation (e.g., catching, chasing and blocking the prey^71^^,^^72^^,^^73^). Such effective spatial and temporal coordination can require advanced theory of mind inference,^74^ action coordination, and shared intentionality, ultimately reinforcing cooperation.^75^ Together, these competitive and collaborative contexts illustrate how others’ presence reshapes classical foraging trade-offs. We next review the socio-cognitive mechanisms that both enable individuals to navigate such environments and that emerge as consequences of social foraging.

## Socio-cognitive elements of social foraging

In this section, we review important socio-cognitive mechanisms that have been highlighted in studies related to social foraging. We also emphasize that social foraging paradigms can advance investigations into many of these elements, which are crucial to social behavior beyond foraging.

For an individual forager, the decisions about how to find resources depend on the conditions of their external environment, such as resource distributions, costs of travel, and predation threat. As outlined in the previous section, in social foraging, decisions are affected by the context of social interactions, with foragers adjusting their decisions and behaviors in response to the social environment. This dynamic can also give rise to group-level phenomena such as coordination and collective behaviors, which are driven by individual-level mechanisms such as social inference. In many scenarios, social foraging can introduce greater complexity, requiring advanced socio-cognitive elements to make optimal foraging decisions that guide coordination and social learning. However, in other cases, simpler heuristics can leverage social learning or collective behavior without the need for complex socio-cognitive mechanisms.

We review selective general elements of social behavior that are particularly relevant in social foraging, either as drivers of foraging decisions or their emergent consequences. These elements can build on each other and lead to a spectrum of social foraging scenarios, ranging from minimal interactions to collective behavior. For example, having a theory of mind can provide a foundation for coordination and social learning, which can be essential for collective behavior. We outline these elements separately, but decisions in social foraging are likely driven by an integration of these processes^76^ (see next section).

### Individual-level drivers of social foraging

#### Behavioral flexibility and dynamic updating

Two key features underlie many social decisions in foraging: flexibility and dynamic updating. To deal with rapidly changing task conditions, decisions and the processes underlying the decisions need to be flexible. Physical environments are mostly predictable and change over slow timescales. However, the addition of other foragers—each with their own goals and decisions—transforms the decision landscape from a relatively stable terrain into a constantly shifting “dynamic seascape”.^77^ Navigating environments with other agents, whose behaviors and intentions can change, demands faster and more flexible decision-making.^78^ Successful social foragers must continuously monitor others’ behaviors and goals, while updating their actions at a pace that matches the rapidly evolving social context, in both competitive and collaborative contexts. This social complexity necessitates rapid cognitive processing across multiple domains—perception, learning, memory retrieval, and decision execution—potentially engaging neural circuits distinct from those involved in non-social behaviors.^79^ The uncertainty of the social environment can also require individuals to develop and apply various socio-cognitive processes^78^^,^^80^ to interpret and respond to their environment and the social dynamics that unfold from these interactions.

Recent studies with naturalistic paradigms have highlighted that individuals apply information about others’ actions in dynamic and instantaneous ways that can substantially affect their decisions and the emergent dynamics of coordination or leader-follower strategies.^17^^,^^43^^,^^81^ In a recent study, Lewen et al.^82^ developed a social foraging task where dyads navigated in a continuous shared space to collect rewards within patches in real time. They found that dyads spontaneously converge upon stable strategies, ranging from cooperative to intermediate, to competitive, and that these strategies are based on dynamic factors related to choice histories, movement costs, and sensorimotor communication. Many of the examples in the following subsections highlight the prevalence of flexibility and dynamic updating in social foraging.

#### Social inference: Theory of mind

Given the complexity of a social environment, it can be essential for individuals to interpret and predict the intentions, beliefs, and motivations of their foraging partners, or at the very least, predict their actions. Cross-species research demonstrates that the degree of sociality in a species correlates with its capacity to develop a theory of mind—the ability to represent others’ beliefs and intentions. Some deeply social primates utilize behavioral features such as gaze or movement direction to infer others’ goals and to modify their own behavior.^83^ Interacting with others can also engage recursive thinking about others’ mental states (what X thinks that Y believes) (see Rusch et al.^84^ for a review), and these representations must be updated dynamically as social situations update.^85^^,^^86^^,^^87^

During social foraging, especially with continuous movements and environmental uncertainty, the representation of others’ beliefs can be necessary for efficient foraging. For example, pursuing a risky option is optimal if and only if more foragers are joining, which lowers the risk of those options (like the stag in Figure 1). Therefore, before making a foraging decision, an individual’s ability to infer others’ intentions and actions can facilitate downstream decisions, such as choosing which patch or prey to pursue or whether to cooperate or compete. A spatial social foraging task, modeled on the stag-hunt game, showed that people engage in rapid online assessment of others’ actions and intentions and that such recursive reasoning about others’ actions drives cooperation.^75^ Another foraging task further demonstrated that inferences about others’ intentions enable flexible shifts between cooperative and competitive strategies as needed.^88^ In sum, knowledge about other agents’ preferences and goals is critically important for survival and foraging decisions^89^ and social foraging paradigms offer an ecological framework to test these sophisticated social computations.

#### Group identity

Social inference may not be necessary to infer cooperative or competitive intentions if individuals can rely on social heuristics like group identity. Social categorizations shape not only whether individuals choose to cooperate or compete but also the underlying psychological processes and motivations driving their foraging decisions. A social foraging experiment^90^ had participants search for hidden eggs in a room where mutual assistance could increase rewards. Results demonstrated that individuals who shared group membership showed higher levels of cooperation and achieved greater foraging efficiency compared to outgroup pairings. Importantly, ingroup foraging was characterized by shared intentionality and mutual representation of goals, while outgroup foraging typically featured more self-focused, individualistic goal orientations.

Working with ingroup members vs. outgroup members can elicit different neural computations. A study, where participants had to make trust decisions regarding investing rewards in ingroup or outgroup members, showed that trusting ingroup members elicits neural activity associated with reward, while trusting outgroup members engages brain regions associated with top-down control and cognitive difficulty.^91^ This finding suggests that in-group cooperation is based on subjectively valued trust, while out-group cooperation requires more effortful and deliberate control, which can diminish under pressures of time or cognitive load. Beyond individual interactions, intergroup competition and perceived outgroup threats can fundamentally alter group dynamics. For example, chimpanzee hunting groups exhibit increased cohesion, spatial proximity, and prosocial behaviors when facing outgroup threats.^92^ Social foraging paradigms, therefore, can help to investigate how group identity influences critical social processes, including competition, trust formation, and cooperative behavior, and how it interacts with other costs and benefits of foraging decisions.

#### Social learning

When foraging alone, individuals must learn about the environment’s resources and dangers through direct experience, which often carries considerable risk. In contrast, group foraging enables social learning, allowing individuals to acquire environmental knowledge from others, while avoiding personal costs associated with learning.^34^ Social learning can guide foraging decisions discussed in the previous section, like when to depart a patch and which patch to choose or avoid, and can be at play in both competitive and collaborative contexts. Solitary foragers must continuously assess patch quality while foraging, whereas group foragers can leverage others’ estimations to evaluate patches more efficiently.^93^^,^^94^ They can also infer the hidden reward structure of the environment from observing others’ behavior.^95^^,^^96^ An experiment where a spatially correlated multi-armed bandit paradigm was used to simulate foraging showed that participants learned the latent, generative rules underlying patterns of resource distribution through social observation.^97^

Although foragers can directly copy others’ actions to learn where to seek rewards and where to avoid threats, effective social learning requires attending to salient information about foraging partners^98^ and using social information strategically. Selective social learning can be especially necessary when social information may be unreliable, inaccurate, or non-generalizable^95^ and can lead to maladaptive outcomes.^63^^,^^99^ Effective social learning can be driven by social inference about partners’ actions through mentalizing about others’ preferences, skills, or goals.^16^ Foragers can also rely on social learning heuristics (or social learning strategies) and choose to selectively copy based on rules like others’ success (“copy the successful”) or behavior popularity (“copy the majority”).^100^ For example, the presence (or absence) of others on a patch can be a valuable source of information about the patch’s quality. Goldstone et al.^101^ showed that when people could see which patch others preferred (a signal of patch quality), they chose those patches despite the risk of overcrowding. Heuristics such as copying the successful or the majority can provide indirect measures of decision quality, enabling strategies that are likely to succeed^102^ at reduced computational costs.^103^

Social foragers can also flexibly apply social learning based on state-dependent factors like their own success rate. In a recent study, Wu et al.^94^ designed an immersive social foraging experiment based on Minecraft and showed that people adaptively decide whether to forage away from, or near to others, and make decisions about whom to forage with. They found that people continually integrate individual reward rates with others’ successes to make these decisions: following successful people when foragers are unable to find rewards by themselves. Models and experimental tasks based on social foraging have increasingly demonstrated that optimal social learning strategies are substantially influenced by both the environmental structure and the characteristics of social partners.^63^^,^^94^^,^^104^^,^^105^

### Group-level consequences of social foraging

Foraging decisions of multiple individuals can give rise to group-level phenomena, such as social network formation, coordination, and collective intelligence, which in many cases rely on socio-cognitive processes like theory of mind or social heuristics.

#### Coordination

When two or more individuals interact to make foraging-related choices like patch selection or exploration, they may need to coordinate with others to achieve individual and group goals. Coordination becomes especially adaptive when individuals face multiple costly and mutually exclusive activities. Since foraging demands significant investment of personal resources (in terms of effort, energy, and time), simultaneous allocation of resources to other crucial activities, such as predator vigilance, can deplete a forager’s physical and mental reserves.^106^^,^^107^^,^^108^ Coordinating with group members can help distribute tasks among the group members and improve efficiency. Such coordination becomes more critical in complex activities like hunting, where success depends on predicting both the prey’s movements and the fellow hunters’ actions.^75^^,^^109^^,^^110^ The benefits and costs of coordination are further shaped by socio-ecological factors; e.g., the presence of shared risks^111^ and benefits^112^ among foragers and a low success rate of solitary foraging^113^ can motivate the emergence of coordination.

Mechanisms such as social inference pave the way for coordination. Studies have shown that coordination can require an iterative theory-of-mind to infer others’ intentions and actions,^75^^,^^114^ especially to predict their actions before acting themselves.^17^^,^^115^ Several cognitive mechanisms can further facilitate effective coordination, including joint attention,^116^ shared goals, and adherence to social norms.^117^ However, coordination does not always require sophisticated cognition; even simple heuristics such as tit-for-tat^118^ and prosocial turn-taking^81^ can yield stable collaborative dynamics. In addition, a computational model of collaborative hunting leveraging deep reinforcement learning showed that decisions based on simple encoding of distance between an agent and others (as opposed to specific locations) and reward sharing, without the use of theory of mind, can lead to the emergence of complementary rules in hunting.^69^ Thus, social foraging paradigms, especially tasks with rich spatiotemporal affordances, have increasingly shed light on mechanisms of coordination and may help explain how low-level socio-cognitive mechanisms can give rise to complex social behavior.

#### Social structures and relationships

Interacting in a social world can naturally give rise to social networks, which can shape the information available to individuals, their decisions, and their collective behavior. A recent study on social networks of a forager community showed that social networks help share information (or knowledge relevant for foraging) over both short term (such as food locations) and long term (such as foraging skills).^119^ In spatial foraging, the spatial decisions foragers make—regarding movement patterns and patch selection—influence interaction opportunities and consequently mold social networks over time.^94^^,^^120^^,^^121^ Relative differences in foraging behavior and preferences between group members can affect their social network positions. For example, fish that react faster to threats tend to occupy frontal or central positions and thus wield a stronger influence on the group’s escape route.^122^ An individual’s position in a social network can directly affect their foraging efficiency, too. In birds, individuals who are centrally located in a network have been shown to have better access to social information and be more likely to discover novel patches.^123^ Additional animal studies have revealed how foraging behavior is shaped by the dynamic interplay between social network structure and group composition, specifically, the diverse characteristics of individual group members.^124^

To operate in a dynamic social world, individuals need to mentalize not only others’ preferences or beliefs but also the social relationships between other individuals. Recent research with social animals has highlighted how foraging interactions are guided by individuals’ mental representations of their social relationships and group structure. For example, studies with bats show that foraging interactions induce neural synchronization (inter-brain coupling) that varies in strength based on the individual’s position within the social network.^125^ Foragers not only monitor their direct interactions but also track relationships between other group members,^126^ integrating this information into their foraging decisions. In human studies, economic paradigms demonstrate that individuals weigh social information differently depending on network structure^127^ and that the social network structure can, in turn, affect collective dynamics like the spread of cooperation.^128^ Integrating approaches from network science with social foraging research offers promising opportunities to uncover how social network topology influences foraging decisions, and vice versa.

#### Group decisions and collective intelligence

Social foraging affords an opportunity for individuals to act collectively*,* i.e., as a group. Coupled social interactions can give rise to collective intelligence that exceeds the capabilities of any individual.^129^ Opportunities for information exchange between foragers can enable coordination^130^^,^^131^ and cooperation (as shown in hunter-gatherers^132^). Collective decisions about where and when to move can increase a group’s accuracy and performance.^133^ Collective decisions can emerge from simple, individual-level patch-choice rules that create positive feedback loops. In birds, for instance, seeing conspecifics at a patch increases the probability of joining and each additional joiner boosts that probability non-linearly.^134^ Such cascading decisions can rapidly generate group-level aggregation and synchronized foraging, and further flexibility in such simple rules of attraction based on the context can drive adaptive collective decisions.^63^^,^^135^ Individual-level decisions based on costs and benefits can also affect group-level outcomes, such as group movement and cohesion (see Conradt and Roper^136^ for a game-theoretic perspective to the emergence of group decisions).

A group’s collective efficiency depends on multiple interacting factors: the structural features of the environment,^94^ the diversity and distribution of knowledge and skills among group members,^137^ group composition,^138^ the alignment of individual and group incentives,^139^^,^^140^ and the patterns of interactions between group members.^63^^,^^141^ The presence of many other individuals in a group can also boost collective sensing and distributed processing. In humans, larger groups, aided by flexible social inference can boost the search for a hidden reward in a spatial task.^104^ However, in other contexts and species, such collective intelligence can emerge from relatively simple individual-level heuristics.^142^

Other experimental research on human collective foraging shows that efficiency in collective performance is driven by flexible adaptation of foraging and social learning strategies.^94^^,^^140^^,^^143^ In a recent study, Deffner et al.^140^ designed an immersive collective search experiment and showed that selective use of social information improves performance. Theory of mind can further lead to collective intelligence through efficient coordination that promotes self-organization into complementary or synergistic roles.^114^ Integrating foraging theory with models of collective behavior presents a promising direction for future research, offering testable predictions about how groups optimize collective performance across diverse contexts. Optimal foraging theory, like the marginal value theorem, can make predictions about how groups resolve conflicts over when to leave a foraging patch^144^ and decisions regarding collective movement.^145^

## Social foraging: An integrative and unifying framework

Based on the review of social foraging-related literature and recent studies, a few effects become clear. Social foraging modifies foraging decisions associated with risk and reward and the optimal decisions can be based on the competitive or collaborative context (Figure 1). It introduces new decisions that are uniquely social, such as who to learn from or who to forage with. Last, socio-cognitive mechanisms like theory of mind and social learning can drive decisions in social foraging and give rise to group-level phenomena like coordination. We now introduce a social foraging framework that can organize and integrate these various concepts and contextualize them within established decision-making frameworks.^146^

**Figure 2:**
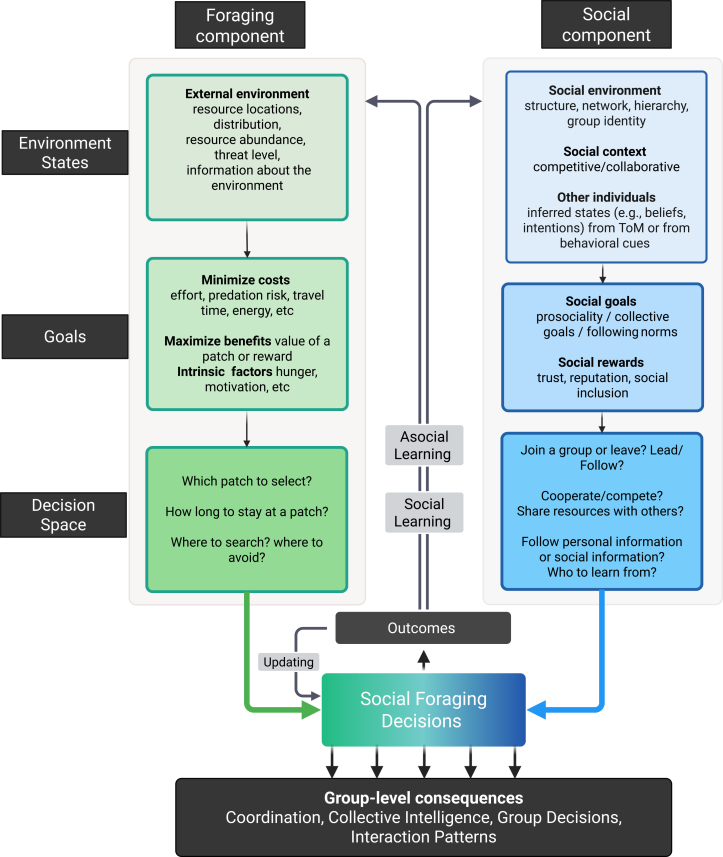
Social foraging framework Social foraging decisions can be broken down into two components: foraging (left) and social (right). Within each component, we further distinguish between *environmental states* (i.e., the external states like reward distribution or social structures); g*oals* of an agent and the possible actions available in the *decision space*. These processes jointly inform social foraging decisions through an integration function that weighs different goals to select an action. Agents can then update external states and goals via *asocial learning* and/or *social learning* based on the outcome of the selected action (which may depend on others’ actions). Agents can also avoid costly computations associated with such learning by updating the value of a decision based on its observed outcomes, at the cost of reduced flexibility (shown by the arrow “updating”). Multiple agents making such decisions (shown as multiple parallel arrows) can give rise to *group-level consequences* (e.g., coordination, collective intelligence), which feedback into the social environment.

We conceptualize the social foraging process as a combination of two interrelated components: an asocial *foraging* component and a *social* component (Figure 2). The asocial foraging component characterizes the commonly studied decisions that an individual is confronted with regarding foraging activities like finding food or avoiding predators. The social component conceptualizes the unique additions of being in a group and engaging with others, and overlays the foraging component by adding new environmental variables, goals, and expanded decision options. For illustrative purposes, and to emphasize the differences between the asocial and social components, we represent them to be separate in Figure 2. However, we emphasize that social foraging decisions are driven by an integration of both components.

Our framework has a two-fold objective. The first is to organize key concepts from classical social foraging theory and from various studies across disciplines that have leveraged paradigms like social foraging. Second, we highlight the differences between social and solitary foraging and describe how they influence social foraging decisions. We note that this framework is intended as a high-level organizational structure that captures the key components involved in social foraging decisions. While it does not explicitly depict all possible interactions between environmental states, goals, and decision processes, it provides a foundation from which testable predictions can be derived when focusing on specific components. We offer a formal account of the framework in Box 2 to showcase one of the ways to model social foraging decisions and some predictions for the main contexts of social foraging discussed so far. We also note that such computations (detailed in Box 2) may not be necessary in all cases. Simple value-based learning of the decision outcomes and heuristic decision-making can achieve satisfactory outcomes as well, while trading computational efficiency for decision flexibility and generalizability.^103^

### Environmental states

Foraging decisions are informed by the known state of the physical environment, like resource distribution or predator behavior. The social component is driven by the social environment, including information about social partners, their beliefs, knowledge, and intentions, as well as the structure of the social environment, such as social network positions and dominance hierarchies.^125^^,^^126^ Representations of the social environment may be constructed and revised through direct observation of others’ behavior (e.g., presence, proximity, and movement patterns) or via theory of mind—inferring others’ unobservable mental states such as beliefs and intentions. These representations can be updated through learning mechanisms that integrate perceptual, contextual, and inferred social cues. Social and nonsocial environmental states are distinct but not independent. For example, in a collaborative social environment, individuals may perceive reduced risk in the foraging environment, whereas in a competitive environment, individuals may perceive increased scarcity in resources. A social environment can be richer, more dynamic, and more uncertain than a physical one, where other individuals’ actions can change frequently and their intentions to cooperate or compete can be difficult to infer and may change momentarily.

### Goals

A forager’s primary goal is to maximize their energy returns against the costs of effort or time,^1^^,^^147^ which can be further affected by intrinsic factors like hunger or motivation for seeking rewards.^148^ When foraging in a group, social rewards can be critical for survival, and thus a forager needs to account for social goals like coordination and resource sharing that can accrue social rewards, too.^24^^,^^149^ A weighted integration of foraging and social goals shapes social foraging decisions. The relative importance of different goals can depend on current needs and context^94^; e.g., in collective foraging, the collective or social goals can supersede other individual goals.^17^^,^^140^

Social rewards can outweigh individual energetic returns, where gaining reputation and being socially included in a group may hold more value than immediate returns. The time horizon of social goals can be longer than that of solitary foraging; a social forager may need to give up short-term energy-maximizing goals to achieve long-term benefits (i.e., stabilize benefits over time rather than maximize them). Anthropological and ethnographic findings have shown that reputation for generosity or pro-sociality can be a key driver for cultivating trust between individuals and establishing cooperative partnership, ultimately leading to greater social benefits.^150^ Features of the social environment such as network density and group size can shape social goals too.^151^^,^^152^ For example, densely connected social networks can speed up the flow of social information, which can further increase the value of reputation. Differences in social environments or cross-cultural differences can further affect how people weigh certain social rewards like reputation or trust and trade-off individual vs. group goals.^152^

### Decision space

Foraging decisions revolve around where to search, which patch to exploit, when to leave a patch, and the trade-offs between different choices. Being a part of a group expands the decision space by adding social dimensions, such as coordination and competition, to individual foraging choices. Foragers also need to make decisions related to how to engage with others. For example, they face additional trade-offs like deciding between following others toward a location or not, who to follow, whether to share resources or not, to compete or cooperate, forage for others or not, or which social role to take. High-level social decisions or context like competition or cooperation can affect other decisions in a hierarchical manner^153^ and constrain subsequent foraging decisions. For example, foragers within the same group often collaborate, and frequent collaboration can encourage decisions to share resources, which can, in turn, strengthen social bonds and facilitate decisions to collaborate in future.

### Learning

While a solitary forager can only learn about the environment by trial and error, a social forager can socially learn from others about both the physical and social environment to guide goals and decisions. In contrast to asocial learning, social learning may also occur through social rewards or punishment imposed by others, which can lead to evaluative feedback and formation of social norms.^154^^,^^155^ Social learning via social rewards or punishment can not only allow learning of the correct action but also enable the inference of others’ mental structures, which can provide richer details about the environment.^155^

Both social and asocial learning can occur at the same time to guide social foraging decisions. A forager can infer the underlying value of a patch or a social partner by combining asocially and socially learned information. Representations of the social environment, via theory of mind and flexible updating, can further facilitate this process.^16^^,^^94^ Heuristics (e.g., "copy the majority", and "follow the most successful") and norms^156^ can avoid costly computations underlying theory of mind. Asocial learning about the environment can guide arbitration between social learning mechanisms, such as imitation and emulation, and domain—general reward learning^157^ can drive learning of social features and flexible use of social learning strategies. Furthermore, learning about the environment may not always be necessary for making foraging decisions. Simpler and computationally cheap updating (or model-free learning) based on an action’s outcomes can drive future decisions,^103^ where foragers can learn the value of each strategy from the outcome it yields and repeat the most rewarding action.

### Integrated social decision

A solitary foraging agent makes decisions based on the environmental state and goals and updates them through learning. We argue that a social foraging agent makes decisions based on a representation of the social and foraging environment and selects actions that optimize their social and foraging goals. Thus, decisions in social foraging are a result of an integration of both the asocial foraging and social components.

Social foraging decisions are shaped by the demands of maximizing foraging efficiency, adjusting decisions based on the social environment, and maintaining favorable social outcomes. On one hand, physical environmental states and foraging goals can give rise to social goals. For example, grouping or cooperation becomes more likely under high levels of environmental risk and uncertainty.^10^^,^^59^^,^^158^ Silston et al.^10^ showed that people weigh others’ presence and external threats to evaluate the appropriate patch to select. In the absence of threat, they prefer patches with fewer competitors, while with high levels of threat, they prefer patches with others. In a patch foraging experiment,^159^ researchers showed that foraging in a risky and abundant environment increases resource sharing between people, while they rarely develop sharing norms in environments that are stable. Another recent foraging study showed that people are more likely to forgo collecting rewards for themselves and choose to forage for others in poor environments than in rich ones because of reduced opportunity costs in poor environments.^160^

On the other hand, perceived social environment and its accompanying social goals can suppress foraging goals. For example, people forgo short-term energy gains to share food with others when the sharing norm is present.^161^ Other social environmental factors, such as one’s social position in the hierarchy, can interact with decisions to collect rewards for self or others. Dominant rhesus macaques tend to make more prosocial decisions but only when their decision ensures a reward for themselves too.^162^ These effects are likely to be state-dependent, as well as contingent on others’ behaviors and coupled dynamics between individuals.^163^ At a more basic level, social information can guide decisions such as which patch to select,^164^ how much effort to apply,^165^ or which movement strategies are most effective.^54^^,^^89^^,^^140^

Finally, decisions made by social agents in a group can integrate to influence group-level consequences such as coordination, social networks, social norms and structures, and collective outcomes. These group-level consequences can then feedback into and update social and physical environmental states and foragers’ goals. For instance, one’s decision to cooperate can encourage others to reciprocate cooperation. Prosocial decisions like resource-sharing made by an individual can affect the likelihood of others associating with them and thereby the wider interaction patterns. A study with Maru Hunters showed that an individual’s propensity to share a higher proportion of their income is associated with their centrality in the cooperative hunting network, i.e., the people who share more tend to be preferred partners for cooperative hunting.^150^ In addition, aggregation of individual decisions (like patch discovery) can influence other individuals’ decisions (to exploit others’ discoveries rather than explore), and subsequently the utility of those decisions (if too many foragers exploit rather than explore, group productivity would decline and the utility of exploiting would decrease).

Integration between asocial and social components may be driven either by overlapping computational mechanisms or by different specialized mechanisms that eventually integrate to guide behavior. There is growing evidence for overlap between how we represent spatial and social environments and navigate through them.^166^ Information about others’ positions relative to each other in a social network is represented in a similar fashion as information about physical locations in space.^167^ Social rewards and social threats appear to elicit similar neural representations as foraging-based rewards or predator-based threats.^168^ However, other evidence suggests specialized mechanisms underlying social processes. Social dilemmas have been argued to be driven by two neural networks: a cognitive control system that processes external incentives and a social cognition system that processes trust signals.^169^ In an experiment where participants had to locate a hidden reward under social and asocial cues, social cues were processed in a different manner than asocial cues: people processed social cues based on interaction history and placed more weight on them than they did on asocial cues.^170^ There is also evidence for different neural mechanisms underlying social and asocial learning^171^ and that the brain then arbitrates between these learning strategies based on their relative uncertainties.^172^ Even at the computational level, learning processes in social contexts can differ from those in asocial contexts and need additional accounting for others’ actions.^86^^,^^173^

The social foraging framework, with its ecological and evolutionary lens, can further investigations into the integration between the asocial and social components of decision-making. For instance, it can provide tractable ways to discern how social and asocial environments are processed, represented, and learned—whether through distinct pathways or through similar mechanisms—and how social and asocial goals shift the salience of the two environmental states in the decisions made. It also allows for testable predictions by treating environmental and social features as parameters that dynamically influence goal weighting (see Box 2). Features such as resource scarcity can increase the weight of foraging goals, particularly in contexts where short-term gains are critical. However, in environments where future social returns are expected, such as reciprocal exchange or cooperative hunting, the same scarcity may increase the weight of social goals, encouraging coordination or prosocial behavior. Similarly, social features like group identity, value of reputation, or network position may further shift decisions toward socially beneficial actions, especially when these features enhance observability or expectations of reciprocity. These contextual modulations can also shape the mechanisms that represent the social environments. Competitive interactions may require mentalizing about others’ goals, while collaborative interactions may require mentalizing about others’ actions to coordinate better. Further investigations can test how differences in environmental complexity (e.g., rich visual-spatial foraging vs. abstract binary choices, multiple foragers vs. a dyad) affect the social heuristics or processes employed by foragers.

Box 2 **Social foraging framework: A formal account**

We first consider a solitary agent. An agent’s foraging decisions can be represented by a set of environmental states, *S*_*f*_ = *s*_1_,*s*_2_, …,*s*_*n*_, goals *G*_*f*_ = *g*_1_,*g*_2_, …,*g*_*m*_ over a set of possible actions that an agent can take *A*_*f*_ = *a*_1_,*a*_2_, …,*a*_*k*_. We can now consider a social foraging agent, whose actions will be defined by the foraging component, layered by a social component. A social agent’s environmental states will include *S* = *s*_*s*1_,*s*_*s*2_, …,*s*_*sn*_,*S*_*f*_, goals *G* = *g*_*s*1_,*g*_*s*2_, …,*g*_*sm*_,*G*_*f*_ over a set of possible actions that an agent can take *A* = *a*_1*s*_,*a*_2*s*_, …,*a*_*s*_,*A*_*f*_. The policy function of a social agent, π, can select a decision based on different weightings of foraging and social goals.

The value function for a particular goal *g* is given by $Q^{\pi }\left(s,a|g\right)=\sum_{s^{\prime }}P\left(s^{\prime }|s,a,g\right)\left[U\left(s^{\prime },s,a|g\right)+\gamma \;\max_{a^{\prime }}Q^{\pi }\left(s^{\prime },a^{\prime }|g\right)\right]$, where *P*(*s*′|*s*,*a*,*g*) denotes the probability of transitioning from state *s* to *s*′ given action *a* and goal *g* and γ is the discount factor for future rewards. The overall value function integrates across goals with variable weights:

$$Q^{\pi }\left(s,a\right)=\sum_{i}w\left(g_{i}\right)Q^{\pi }\left(s,a|g_{i}\right)\text{.}$$

The utility function balances self-interest against social considerations:

$$U\left(s^{\prime },s,a|g\right)=\theta \left(g\right)U_{self}\left(s^{\prime },s,a\right)+\left(1-\theta \left(g\right)\right)U_{others}\left(s^{\prime },s,a\right)\text{,}$$

where θ(*g*) represents the goal-specific balance between self and others’ utilities.

The optimal social foraging decision is given by:

$$\pi_{*}\left(s\right)=\arg\max_{a}Q^{\pi }\left(s,a\right)\text{.}$$

We can consider four contexts and predict how environmental states and goals shape decisions.

**Prediction 1: Competitive context.** When an agent is foraging with others for limited resources, without any social goal like gaining reputation, the foraging goal to maximize rewards will guide decisions. The expected utility of an option, like a patch, can be based on social information such as others’ presence, but the utility will be only for the self (θ = 1). The agent needs to consider others’ actions when calculating their own expected energy gain.

**Prediction 2: Mixed collaboration-competition context.** The weight of social goals like gaining reputation or prosociality can be integrated with foraging goals to influence decisions. Foraging environmental features like travel costs or scarcity, and social environmental features like inferred intentions, group identity, or norms can shape the relative weight of the goals. Social and foraging goals may interact and constrain each other rather than independently contributing to behavior. *w*(*g*_*i*_) can also capture the adjusted weight of each goal based on the agent’s internal state and interactions between goals if the goals are conflicting. Agents may update the weight of each goal based on the outcomes, i.e., they may only try to cooperate if they infer an intention to cooperate from others. The discount parameter *γ* can represent goal-specific discount factors and different time horizons for social and foraging goals. Social goals (e.g., maintaining reputation) may have larger *γ* values (longer time horizons) than immediate foraging goals.

**Prediction 3: Collaborative context.** Foraging in a collaborative context, such as with in-group foragers, can increase the weight of social goals like coordination. The agent may assign different utilities to its own outcomes and those of others and may forgo self-benefit to achieve coordination. Achieving coordination may require modeling others’ behavior, and the value functions reflect not only environmental rewards but also social rewards and inferred social contingencies, including expectations about others’ future actions. In the case of stable collaborations, mentalizing other agents’ state may not be necessary to guide decisions, and simpler heuristics could suffice.

**Prediction 4: Collective context.** To illustrate an extreme case of the collaborative context, we can assume that every agent shares the same goal of maximizing group benefits. We can assume that a shared collective goal *g*_*collective*_ and collective utility *U*_*collective*_ drive decisions. We can also assume that the weights of other goals will depend on *g*_*collective*_ and can vary to influence the correlation between agents’ weights. A weight function like *w*_*homogen*__*e*__*ous*_(*g*_*i*_|*g*_*collective*_) would lead all agents to converge toward a similar behavior, maximizing the Q-values that align with the collective goal. *w*_*complementary*_(*g*_*i*_|*g*_*collective*_) would allow for coordination or division of tasks, where different agents prioritize different individual goals that complement each other under the collective goal.

## Discussion

Integrating various strands of research under a broader paradigm has been a long-standing challenge in the behavioral sciences. Newell^174^ advised focusing on a single complex task. Rather than designing specific, small, disparate experiments aimed at specific, small, disjointed questions, he advocated for framing research pursuits around a single complex task. This raises an important methodological question: how can we effectively choose a model or research paradigm to achieve this goal? Biological sciences, where research is usually focused on a model organism, can offer insights. A good model organism should be tractable to study in laboratories, be evolutionarily related to the target of interest, be well-defined in its properties, and be complex enough to spur various lines of investigation.^175^ In many ways, foraging paradigms check these boxes.

Social foraging paradigms can build on the established framework of foraging theory and afford mechanistic insights into social behavior.^36^^,^^176^ Previous accounts of social foraging theory have established formal theoretical models to explain different strategies observed in nature, such as producer-scrounger dynamics and selfish vs. cooperative behaviors. These models, grounded in economic and game-theoretic perspectives, define what is being optimized and why certain strategies emerge under specific conditions. They offer testable predictions about the conditions that favor particular strategies but often leave the underlying mechanisms underspecified. In this article, we have complemented these formal models by examining the socio-cognitive mechanisms underlying social foraging decisions. We synthesized research on how these decisions are implemented through psychological processes such as theory of mind and social learning, and their group-level consequences such as coordination and collective intelligence. As such, we identify social foraging as a unifying framework that can be used to study these underlying processes and enrich the study of social behavior. We present this framework as a flexible scaffold—broad enough to capture diverse aspects of social behavior yet open to integration with more specific theories and mechanisms.

A core assumption of foraging paradigms is that decisions are reward-maximizing, and individual decisions can be understood by evaluating how they weigh the costs and benefits of given choices and context. While this assumption serves as a clear basis for the motivations and computations underlying decisions, the utilitarian bent of this assumption can appear to be limited in its applicability. Satisficing heuristics or social norms are likely to be at play in social foraging decisions, especially given the dynamic and unpredictable nature of many social decisions that can increase computational costs.^177^^,^^178^ Likewise, there are other affective and motivational factors that influence social foraging decisions, such as reputation goals or group identity*.* Our paper has highlighted how simpler heuristics and other factors play a role in social foraging decisions. Therefore, by explicating and accounting for social goals and general social complexities, social foraging can help explain certain behaviors that do not seem optimal.

Social foraging can also appear to be limited in its application to understanding modern human social behavior (e.g., social media). However, it can serve as a baseline model to compare behavior in contexts that have increasingly diverged from those associated with our evolution.^179^ Reward learning, with its roots in foraging, has long been associated with animal behavior and human decisions. Recent studies have shown it to be applicable to behavior on social media.^180^^,^^181^ Similarly, insights from social foraging can be applied to understand how fundamental social decision-making processes interact with the affordances of social media platforms and shape the decisions available to people.

Recent advances in experimental design and computational modeling bode well for the study of social foraging and make it a tractable paradigm. Gamification of experiments^6^^,^^10^^,^^94^^,^^104^^,^^140^^,^^179^ can target rich, naturalistic social behavior with continuous decision-making. Theoretical approaches can employ agent-based models to focus on emergent social behavior or underlying cognitive computations.^54^^,^^63^^,^^105^^,^^120^ Advances in computational modeling of behavioral and neural data can further yield mechanistic insights.^129^^,^^180^ For example, hyperscanning, where two or more individuals simultaneously engage in a task or interact, and their brain activity is simultaneously recorded, can provide insight into the workings of real-time social interactions^181^ or how the social environment is encoded.^182^ Computational modeling of social foraging behavior can also disambiguate individual-level behavior, goals, and decisions from group-level actions and goals, which is necessary to explain how groups achieve collective intelligence.^183^

In conclusion, we live in highly complex social environments and have evolved within them. Foraging theory has helped contextualize adaptive decisions and underlying cognitive processes. Foraging as a unifying framework has helped bridge fields like animal behavior,^184^^,^^185^^,^^186^ human cognition,^187^^,^^188^^,^^189^ developmental psychology,^190^ and psychiatry.^148^^,^^191^^,^^192^^,^^193^^,^^194^ Similarly, social foraging can serve as a thread that ties the study of social behavior, linking its evolution, development, maladaptive manifestations, and cross-cultural differences. It can connect multiple disciplines by offering a common language to drive comparative inquiries.^195^ Social species differ vastly in their group dynamics^196^^,^^197^ and social foraging decisions--driven by social and environmental features at the individual level--can provide further insight into those differences. It can also help extend asocial theories of cognition into the social domain (as we summarize in Table 1), where similarities in asocial and social foraging can pinpoint fundamental computations of decision-making, while differences can reveal unique computational processes and behavioral or neural adaptations.

**Table 1:** Decision-making extensions in social foraging

Aspects of decision-making	Examples from foraging paradigms	Modifications in social foraging paradigms
Exploration vs. exploitation (e.g., patch-residence decision, whether to search for patches nearby or far, whether to continue exploiting the current patch or explore for more)	How do individuals balance exploration and exploitation in patch choices?^7^^,^^198^^,^^199^^,^^200^^,^^201^ How should resources like time, energy, and effort be expended on these activities?	How do competitive or cooperative social contexts affect this trade-off? How do groups manage this trade-off?^54^^,^^63^^,^^140^ How does the presence of others change allocation of time or energy?^165^ How do groups distribute effort among themselves for different activities?^60^
Risk sensitivity (e.g., patch/prey choice decision: whether to select a patch or foraging action associated with greater risk but greater reward or select an option with less reward and less risk)	How do probability distributions of reward affect expected utility and choices? How do individuals balance risks and rewards in foraging?^193^^,^^202^^,^^203^^,^^204^^,^^205^	How does risk sensitivity change as a function of group size?^10^ How do foragers coordinate with others who differ in risk preferences? Do differences in risk manifest in or lead to different social roles?^206^
Learning	How do foragers learn about the properties of the resource environment? Do model-free or model-based learning rules describe foraging decisions?^5^^,^^207^^,^^208^	How do individuals integrate social and asocial learning and selectively learn from others? How does social learning inform movement and social interactions?^94^^,^^140^ How does social context (competition or cooperation) affect learning rates?^209^
Representation of the environment	How do individuals navigate new environments and learn the location of resources or predators? How does spatial memory affect foraging decisions? How do goals affect attention and memory?^210^^,^^211^^,^^212^^,^^213^	How do foragers construct cognitive maps of dynamic social agents? How does social learning or social integration affect spatial memory?^89^^,^^214^^,^^215^
Continuous decisions	How do foraging decisions like when or where to move unfold over time^6^^,^^216^^,^^217^?	How does movement toward social partners unfold over time in naturalistic interactions? How do others’ movements affect decisions?^75^^,^^94^^,^^140^^,^^218^

Foraging paradigms have shed light on important aspects of decision-making. We offer future research questions that can extend these findings in the social context through social foraging paradigms, along with related papers that have addressed similar questions.

We aim to position social foraging as a unifying framework for understanding human social behavior in naturalistic settings, while also promoting the exchange of research questions and insights across species, ultimately advancing our understanding of the general principles of social behavior. We propose that social foraging is a complex “super-task”^219^ that can substantially integrate smaller tasks and make them commensurate among themselves to yield a detailed picture of social behavior.^220^ It can help go beyond the individual and study behavior across scales—from individuals to dyads, groups, and collectives.

**Glossary**

**Optimal foraging theory:** A framework in behavioral ecology that models how animals maximize their energy intake per unit of time by making efficient decisions about what, where, and how to forage.

**Marginal value theorem:** A model in optimal foraging theory that predicts the optimal time an animal should spend in a resource patch before leaving to search for a new one, in order to maximize its overall rate of resource intake.

**Patch-choice model:** A theoretical framework in foraging ecology that describes how animals decide where to search for food among discrete patches or options, often guided by expected value based on costs of search, handling, or risk, and benefits such as energy or rewards.

**Risk dilution:** The reduction in individual predation risk that occurs when foraging in groups, due to the probability that any one individual will be targeted by a predator decreases with increasing group size.

**Information sharing:** Exchange of information between individuals about the physical or social environment. This can occur inadvertently (e.g., a forager’s location unintentionally signals a patch's location or quality to others) or through active communication.

**Competitive social context:** A context of social interaction where individuals or groups compete for the same limited resources, such as food, mates, or space, where one individual’s gain usually reduces the availability of resources for others.

**Collaborative social context:** A context of social interaction where individuals benefit from each other’s presence or from foraging with others. Coordination toward a shared goal or aligned goals can be a special case of collaboration.

**Cooperation:** A form of social interaction where individuals may make decisions that benefit others or the group, even at personal costs. Mechanisms like kin selection, reciprocity, social norms, and group selection can support cooperative behaviors.

**Resource sharing:** The distribution of acquired resources among individuals, which may occur through reciprocity, tolerance, or enforcement mechanisms and often influences social dynamics and foraging outcomes.

**Social learning strategies:** Rules individuals use to acquire knowledge and skills by observing and interacting with others. They guide decisions about “when” to socially learn, “who” to learn from, and “what” to learn.

**Collective intelligence:** The enhanced problem-solving or decision-making capacity that emerges from the aggregation of individual knowledge. It can emerge from simple heuristics, such as distributed sensing or wisdom of crowds, or through more coordinated actions like in human teams.

**Game-theoretic models:** Formal frameworks that analyze strategic interactions between decision-makers (players), where each individual’s outcome depends not only on their own choices but also on the choices of others.

**Producer-scrounger model:** A foraging strategy model where individuals adopt one of two roles: producers, who search for and discover food, and scroungers, who exploit the discoveries made by producers.

## Acknowledgments

K.G. thanks Cecilia Padilla-Iglesias, Cody Moser, and James Brooks for their thoughtful feedback. D.M. is supported by the 10.13039/100000025National Institute of Mental Health grant (R01MH133730-01).

## Author contributions

K.G. and W.D. wrote the initial draft. K.G. and W.D. created visualizations. K.G., W.D., and D.M. edited and revised the manuscript.

## Declaration of interests

The authors declare no competing interests.
